# Remotely sensed indicators and open-access biodiversity data to assess bird diversity patterns in Mediterranean rural landscapes

**DOI:** 10.1038/s41598-019-43330-3

**Published:** 2019-05-02

**Authors:** Inês Ribeiro, Vânia Proença, Pere Serra, Jorge Palma, Cristina Domingo-Marimon, Xavier Pons, Tiago Domingos

**Affiliations:** 1 MARETEC, Instituto Superior Técnico, Universidade de Lisboa, Av. Rovisco Pais 1, 1049-001 Lisboa, Portugal; 2 Grumets Research Group, Department of Geography, Universitat Autònoma de Barcelona, Edifici B, Campus de la UAB, Barcelona, Spain; 3 Grumets Research Group, CREAF, Universitat Autònoma de Barcelona, Edifici B, Campus de la UAB, Barcelona, Spain

**Keywords:** Biodiversity, Community ecology

## Abstract

Biodiversity monitoring at simultaneously fine spatial resolutions and large spatial extents is needed but limited by operational trade-offs and costs. Open-access data may be cost-effective to address those limitations. We test the use of open-access satellite imagery (NDVI texture variables) and biodiversity data, assembled from GBIF, to investigate the relative importance of variables of habitat extent and structure as indicators of bird community richness and dissimilarity in the Alentejo region (Portugal). Results show that, at the landscape scale, forest bird richness is better indicated by the availability of tree cover in the overall landscape than by the extent or structure of the forest habitats. Open-land birds also respond to landscape structure, namely to the spectral homogeneity and size of open-land patches and to the presence of perennial vegetation amid herbaceous habitats. Moreover, structure variables were more important than climate variables or geographic distance to explain community dissimilarity patterns at the regional scale. Overall, summer imagery, when perennial vegetation is more discernible, is particularly suited to inform indicators of forest and open-land bird community richness and dissimilarity, while spring imagery appears to be also useful to inform indicators of open-land bird richness.

## Introduction

Species diversity patterns are shaped by multiple factors, including environmental factors, such as climate, primary productivity, habitat area and habitat diversity, and species-specific factors, such as species functional traits and evolutionary history^1–3^. The relative effect of the environmental factors on species diversity may differ with scale, according to the spatial extent and grain of their gradient of variation^4^. For instance, at large spatial extents, such as continental or regional extents (>100 km), the environmental gradient of climate variables is broader and more pronounced and so are the effects on species diversity^3,4^. Conversely, the effect of habitat variables tends to gain importance at smaller extents, such as landscape or local extents (<10 km), because the gradient of variation is finer and saturates at larger scales^1–4^. Regarding species-specific factors, species with different ecological requirements will show different responses to environmental factors and to environmental changes. For instance, the use of species groups based on habitat preferences may help to unravel different responses to habitat change within species communities^3,5,6^.

Changes in ecosystem area, documented by airborne and satellite imagery, and by the derived map products, have been used to assess human direct impacts on habitat availability and estimate biodiversity change^7–9^. However, the use of area alone as an indicator of habitat change may only partially capture changes in habitat availability for species^10,11^. That is the case when changes at fine spatial grains affect ecosystem structure, but not the main features that define the ecosystem type neither its overall extent. For instance, changes in tree density or vegetation structure, may affect habitat availability, including its quality and structural diversity, within apparently stable forest patches, and consequently affect species presence and diversity patterns^6,12–14^. Moreover, habitat structure is often described by coarse grain metrics of landscape configuration based on the size, shape, and type of land cover patches, thus overlooking variation at finer spatial grains.

Mapping and monitoring ecosystem structure at a fine resolution in large spatial extents poses an operational trade-off, because it requires an intensive sampling effort over a large area^15^. However, in a time of fast environmental change, the assessment of biodiversity response at large spatial extents, fine spatial resolution and frequent time intervals is necessary^16–18^. Satellite remote sensing offers a tool to overcome such trade-off, as it allows to capture variations in ecosystem structure at fine spatial grains across large spatial extents and with high sampling frequency^18,19^. Notably, image texture measures (i.e. measures of the variability of pixel values in a given area) from satellite imagery have been used as a surrogate of vegetation and landscape structure^15,20^, and were found to be a good predictor of species richness patterns^21–23^. For instance, St. Louis *et al*.^22^ show that NDVI (Normalized Difference Vegetation Index) texture measures from satellite imagery were good surrogates of habitat structure and better indicators of bird richness than indices of landscape composition based on land cover maps. Similarly, Culbert *et al*.^24^ and Wood *et al*.^20^ show that NDVI textures measures, used as proxies of vegetation structure, are good indicators of bird species richness. Moreover, in a recent study, in Mediterranean forest habitats, Ozdemir *et al*.^25^ also report that bird richness was best described when using texture measures from NDVI layers than textures of individual Landsat TM bands.

**Figure 1:**
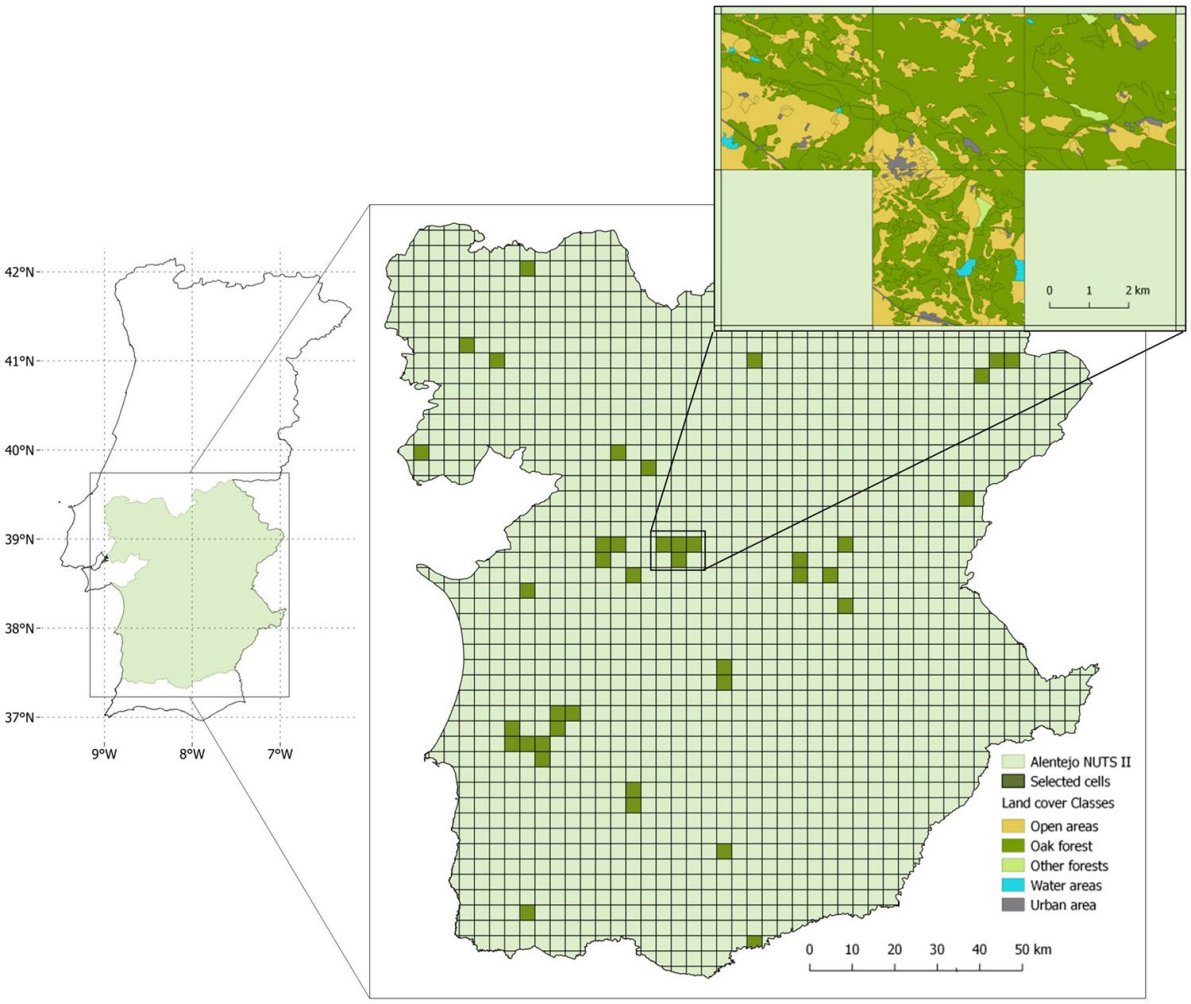
Distribution of the 40 selected cells in Alentejo (i.e., well surveyed cells with eligible land cover, see methods for details on cell selection). An example of the land cover mosaic is shown for four cells.

This study investigates the importance of variables of habitat extent and structure in explaining the patterns of bird species richness and community dissimilarity in the Alentejo region (NUTS II; 31551 km^2^) in Portugal (Fig. 1). This region is characterized by heterogeneous landscapes and seasonal Mediterranean climate. Cropland, oak forest and montados (traditional agro-forestry systems) occupy a large share of the territory, providing key habitats for bird communities^26,27^, and forming landscape mosaics with diverse levels of spatial heterogeneity. This heterogeneity is maintained both by the habitat mix and landscape configuration, at coarser grains, and by the variation in vegetation structure at finer grains. A dataset of bird species occurrences in 40 landscape-sized cells, distributed across the region, was compiled for this study using up-to-date data made available by GBIF (Global Biodiversity Information Facility; GBIF.org^28^), and after checking for data quality (Supplementary Fig. S1). Furthermore, bird data were sorted into two species groups of habitat affinity, that is, forest birds and open-land birds, because of their different ecological requirements.

We expect the richness of a species group to be positively related to the extent and structural diversity of their main habitat in the landscape mosaic (i.e., oak forest for forest birds, open-land for open-land birds), with both factors being more important than the overall landscape structure. That is, species will be more responsive to the features of their preferred habitat, even if they use the whole landscape^5,29^. Moreover, because the overall distribution range of most species in our dataset covers the study area, we also expect habitat extent and structure to be more important than geographic distance or climate gradients when explaining the level of dissimilarity between communities in the study area. Hence, we expect habitat descriptors, including remotely sensed variables, to be good indicators of bird richness at the landscape scale (i.e., the grid cell) and of community dissimilarity at the regional scale (i.e., Alentejo). Moreover, we expect summer satellite data, which provides better information on perennial tree cover, to be a better indicator of forest bird communities, and spring satellite data, which also capture herbaceous cover at its peak productivity, to be a better indicator of open-land bird communities. Finally, open-land birds may show stronger responses to habitat structure variables than forest birds because these variables were derived from optical remotely sensed data, which do not capture understory vegetation structure.

## Results

We applied a multistep protocol to ensure the quality of the bird occurrence dataset prior to data analyses. The final dataset of bird occurrences included 7858 observations of 78 species in 40 landscape-sized cells distributed across the study area (Fig. 1, Supplementary Fig. S1). Our initial list of candidate descriptor variables (Supplementary Table S1) included variables of climate (precipitation, temperature, solar radiation), topography (elevation), land cover (relative cover and largest patch area of selected land cover classes), and image texture. Texture measures were derived from NDVI (a measure of vegetation productivity) and included first-order texture variables (NDVI entropy, NDVI mean, NDVI standard deviation) and second-order texture variables (mean and standard deviation of NDVI entropy or NDVI variance, measured in 3 × 3 and 9 × 9 pixel moving windows). All variables were calculated at the level of the landscape (i.e., grid cell). Second-order texture variables were calculated either for all pixels in the grid cell (i.e., landscape) or for pixels of a focal land cover class (forest or open-land habitats).

From the initial list of candidate variables, we retained a final set of non-collinear variables (Spearman’s rho < |0.7|) per species group (Table 1). Then, we used generalized linear models (GLM) and generalized dissimilarity models (GDM) to investigate the relative importance of the candidate variables in the final sets (Table 1). Finally, the most important variables identified by the GDMs were further tested for their indicator value using a correspondence analysis to test their association with species communities.

**Table 1:** Sets of non-collinear candidate variables per species group.

All Species	rho	Forest bird species	rho	Open-land bird species	rho
%OpnAr	−0.14	%WterAr	−0.15	TmeanT	0.10
NDVI_sd_SP	0.14	NDVI_mn_SP	−0.17	NDVI_var9x9_sd_OP_SP	−0.13
Elev_mn	−0.20	NDVI_ent9x9_sd_OF_SP	0.20	NDVI_ent9x9_mn_OP_SP	−0.14
NDVI_ent9x9_mn_SP	0.20	NDVI_var9x9_sd_SP	−0.21	NDVI_var9x9_mn_OP_SP	−0.15
NDVI_mn_SP	−0.25	%OthFor	0.32	NDVI_var3x3_mn_OP_SU	0.17
%OthFor	0.26	NDVI_var3x3_sd_OF_SP	−0.35	%WterAr	0.22
AnPrecip_mn	0.28	NDVI_ent3x3_sd_SP	−0.37	NDVI_mn_SP	−0.24
RadRg	0.30	%UrbnAr	0.37	NDVI_sd_SP	0.25
NDVI_var3x3_mn_SU	0.36	RadRg	0.40	%UrbnAr	0.32
TmaxJ	−0.38	NDVI_ent3x3_mn_OF_SU	0.53	NDVI_ent3x3_sd_SU	0.35
NDVI_sd_SU	0.38	LgtPtch_OF	0.55		
%UrbnAr	0.48	TminA	0.59		
		NDVI_mn_SU	0.82		

Variables are ordered by the absolute value of the Spearman rank correlation (Spearman’s rho) with the richness of the species group. Climatic (mean annual precipitation - AnPrecip_mn; solar radiation range - RadRg; maximum temperature in June - TmaxJ; minimum temperature in April - TminA and mean trimestral temperature - TmeanT), topographic (mean elevation - Elev_mn) and land cover variables (percentage cover of open land - %OpnAr; percentage cover of urban area - %UrbnAr; percentage cover of water areas - %WterAr; percentage cover of other forest - %OthFor and Largest patch of oak forest - LgtPtch_OF) were measured at the landscape scale (i.e., full grid cell). NDVI variables were measured in spring (SP) and summer (SU) at the landscape scale and at the main habitat scale (i.e., pixels overlapping patches of oak forest (OF) or open-land (OP)). The final sets of candidate variables include first order measures of NDVI mean (mn) and standard deviation (sd) and second-order measures of entropy (ent) and variance (var) in pixel windows of 3 × 3 or 9 × 9. Variables full name is presented in Supplementary Table S1.

### Species richness patterns

The mean NDVI in summer (NDVI_mn_SU) and the radiation range were included in all the best GLMs (i.e., AICc – AICcmin ≤ 2) for forest birds, which suggests that these variables are important descriptors of forest bird richness at the landscape scale (Table 2). Moreover, both variables are positively correlated to forest bird richness (Spearman’s rho = 0.82 and Spearman’s rho = 0.40, respectively) (Table 1). Other variables included in the best models were the standard deviation of second-order NDVI variance in 9 × 9 pixel windows in spring (NDVI_var9X9_sd_SP), the standard deviation of second-order NDVI variance in 3 × 3 pixel windows in oak forest in spring (NDVI_var3x3_sd_OF_SP) and the area of the largest patch of oak forest (LgtPtch_OF) (Table 2). Regarding land cover variables, which are associated with habitat extent, the % cover of oak forest (main habitat) was collinear with LgtPtch_OF (Spearman’s rho = 0.95), and both variables were moderately correlated to NDVI_mn_SU (Spearman’s rho = 0.56 and Spearman’s rho = 0.60, respectively), and to forest bird richness (Spearman’s rho = 0.53 and Spearman’s rho = 0.55, respectively).

**Table 2:** Best generalized linear models of species richness (AICc - AICcmin ≤ 2), the coefficient estimate of the variables included in models are shown (x - variable not included in the model).

							AICc	D^2^
***Forest bird species richness***								
Independent variables	NDVI_mn_SU	RadRg	NDVI_var9X9_sd_SP	LgtPtch_OF	NDVI_var3x3_sd_OF_SP			
Models	43.82	0.03	x	x	x		205.2	0.66
	42.82	0.04	− 0.02	x	x		205.8	0.67
	41.39	0.04	x	x	−0.03		205.9	0.67
	38.13	0.04	x	0.14	x		206.1	0.67
Importance	1.00	0.98	0.34	0.25	0.24			
***Open-land bird species richness***								
Independent variables	NDVI_var9x9_sd_OP_SP	NDVI_ent3x3_sd_SU	NDVI_mn_SP	NDVI_ent9x9_mn_OP_SP	NDVI_var3x3_mn_OP_SU			
Models	−0.04	27.97	−54.39	−1.63	x		220.4	0.46
	−0.04	28.06	−44.39	−1.71	0.01		221.9	0.42
	−0.05	28.39	−52.55	x	x		222.0	0.41
Importance	1.00	0.99	0.81	0.58	0.29			
***Total species richness***								
Independent variables	RadRg	NDVI_var3x3_mn_SU	Elev_mn^2^	NDVI_mn_SP	TmaxJ	Elev_mn		
Models	0.002	0.001	4 × 10^−6^	−1.38	x	−0.001	268.0	0.33
	0.002	x	5 × 10^−6^	−1.30	−0.07	−0.001	270.0	0.30
Importance	0.69	0.65	0.45	0.43	0.35	0.24		

The relative importance across all candidate models is indicated for each independent variable. Variables full name is presented in Supplementary Table S1.

Open-land bird richness, was best described by models including the standard deviation of second-order NDVI variance in 9 × 9 pixel windows in open land in spring (NDVI_var9x9_sd_OP_SP), the standard deviation of second-order NDVI entropy in 3 × 3 pixel windows in open land in spring (NDVI_ent3x3_sd_SU), and the mean NDVI in spring (NDVI_mn_SP) (Table 2). From these, the spring variables are negatively correlated with open-land bird richness, while the summer variable is positively correlated (Table 1). The mean of second-order NDVI entropy in 9 × 9 pixel windows in open land in spring (NDVI_ent9x9_mn_OP_SP) and the mean of second-order NDVI variance in 3 × 3 pixel windows in open land in summer (NDVI_var3x3_mn_OP_SU) were also included in the best models (Table 2). Regarding land cover variables, both the proportion of open-land (main habitat) and the area of the largest patch of open-land were collinear to NDVI_ent3x3_sd_SU (Spearman’s rho = 0.81 and Spearman’s rho = 0.81, respectively). Deviance explained by the best models varied between 66% and 67% for the forest species and between of 46% and 41% for the open-land species (Table 2).

Total bird species richness was weakly described by the best models, with deviance explained varying between 33% and 30%, and no clearly dominant variables (Table 2). The variables included in the best models were radiation range, the mean of second-order NDVI variance in 3 × 3 pixel windows in summer (NDVI_var3x3_mn_SU), mean elevation (including its quadratic term), maximum temperature in June, and the mean NDVI in spring (NDVI_mn_SP). The model-averaged parameter estimates and variable importance of all candidate variables across all candidate models are presented in Supplementary Table S2.

### Species dissimilarity patterns

**Figure 2:**
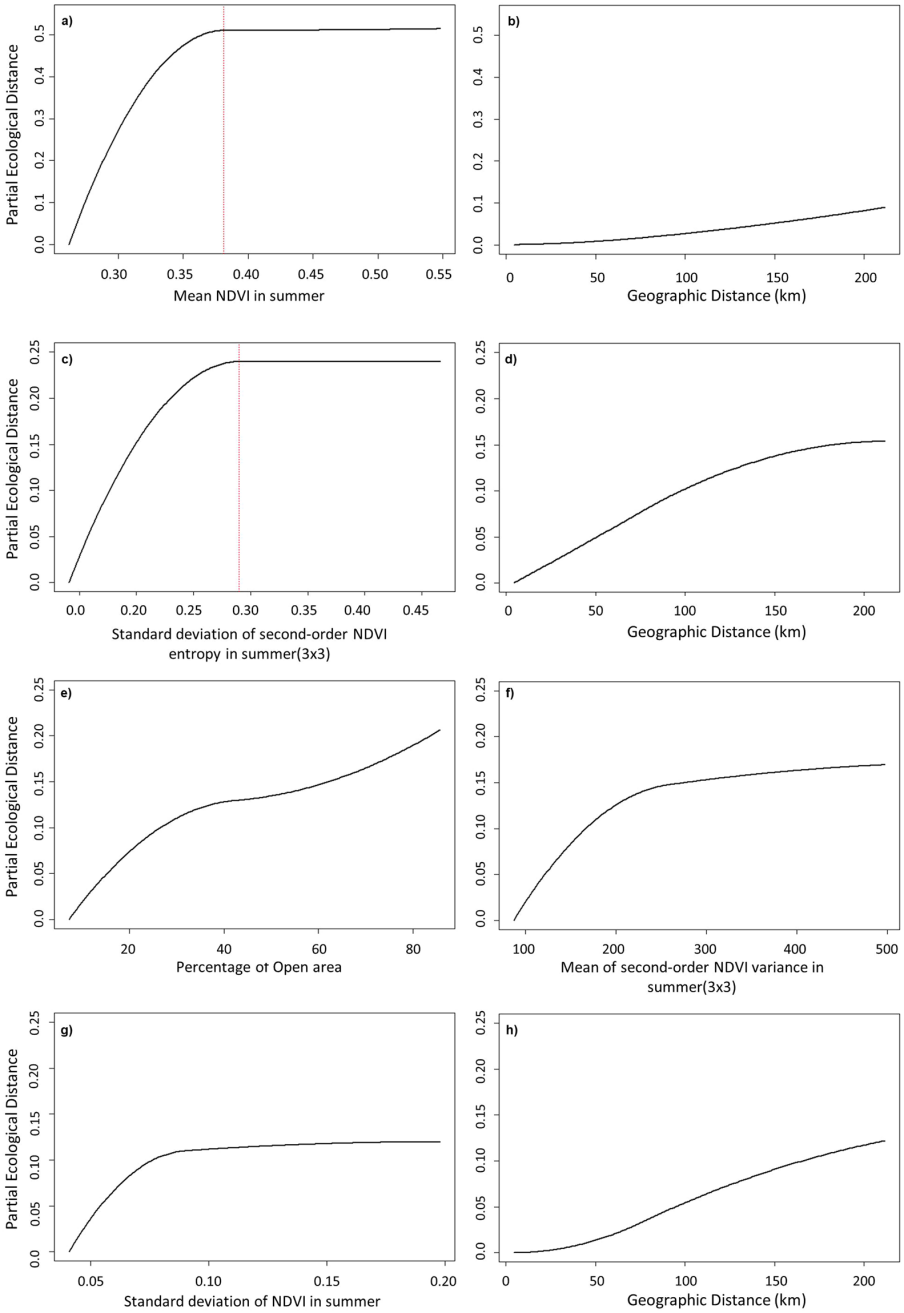
Generalized dissimilarity model-fitted I-splines (partial regression fits) for variables significantly associated with community turnover of forest bird species (**a**,**b**), open-land bird species (**c**,**d**) and all species (**e**–**h**). The maximum height reached by each function indicates the total amount of compositional turnover associated with that variable, holding all other variables constant.

Results from the GDMs suggest that the most important variables explaining species dissimilarity patterns in the study area (i.e., community dissimilarity between landscapes within the region) were, for forest birds, the NDVI_mn_SU and the geographical distance, and, for open-land birds, the NDVI_ent3x3_sd_SU and the geographical distance. For both species groups the compositional changes explained by the changes in vegetation structure, implicit in the gradient of the NDVI variable, mainly occurred below a threshold value becoming negligible above it (Fig. 2a,c). For instance, changes in forest bird communities appear to stabilize above a threshold value for the NDVI_mn_SU, which is possibly related to the availability of arboreal habitat. Also, the NDVI variables, related to vegetation structure, were more important than geographical distance in explaining compositional change (Fig. 2a–d and Table 3), especially for forest birds. The GDM fitted for forest birds explained 36.9% of deviance, while for open-land birds the model explained 23.9% of deviance. The partition of community dissimilarity in the components of species replacement and differences in species richness (Supplementary Fig. S2) suggests that both play a role in explaining compositional differences. Still, species replacement is in general more important for open-land birds, while differences in species richness gains importance for forest birds.

**Table 3:** Significant variables and their relative importance in the GDMs of forest species, open-land species and all species.

	Significant variables	Relative importance	% Deviance explained
***Forest species***			36.947
	NDVI_mn_SU	0.514	
	Geographic distance	0.090	
***Open-land species***			23.874
	NDVI_ent3x3_sd_SU	0.240	
	Geographic distance	0.154	
***All species***			39.844
	% OpnAr	0.208	
	NDVI_var3x3_mn_SU	0.169	
	Geographic distance	0.122	
	NDVI_sd_SU	0.120	

Relative importance is determined by summing the coefficients of the *I-splines* from GDM. The percentage of null deviance explained by the fitted GDM model is also presented. Variables full name is presented in Supplementary Table S1.

The GDM model for all species (39.8% deviance explained) included a land cover variable (% cover of open land, which, in our dataset, is collinear and inversely related to the % cover of oak forest, Spearman’s rho = −0.93), a first-order texture variable (standard deviation of NDVI in summer, NDVI_sd_SU), a second-order texture variable (NDVI_var3x3_mn_SU) and the geographical distance, all of similar importance (Table 3). The partition of community dissimilarity (Supplementary Fig. S2) indicates that compositional changes are mostly explained by species replacement.

**Figure 3:**
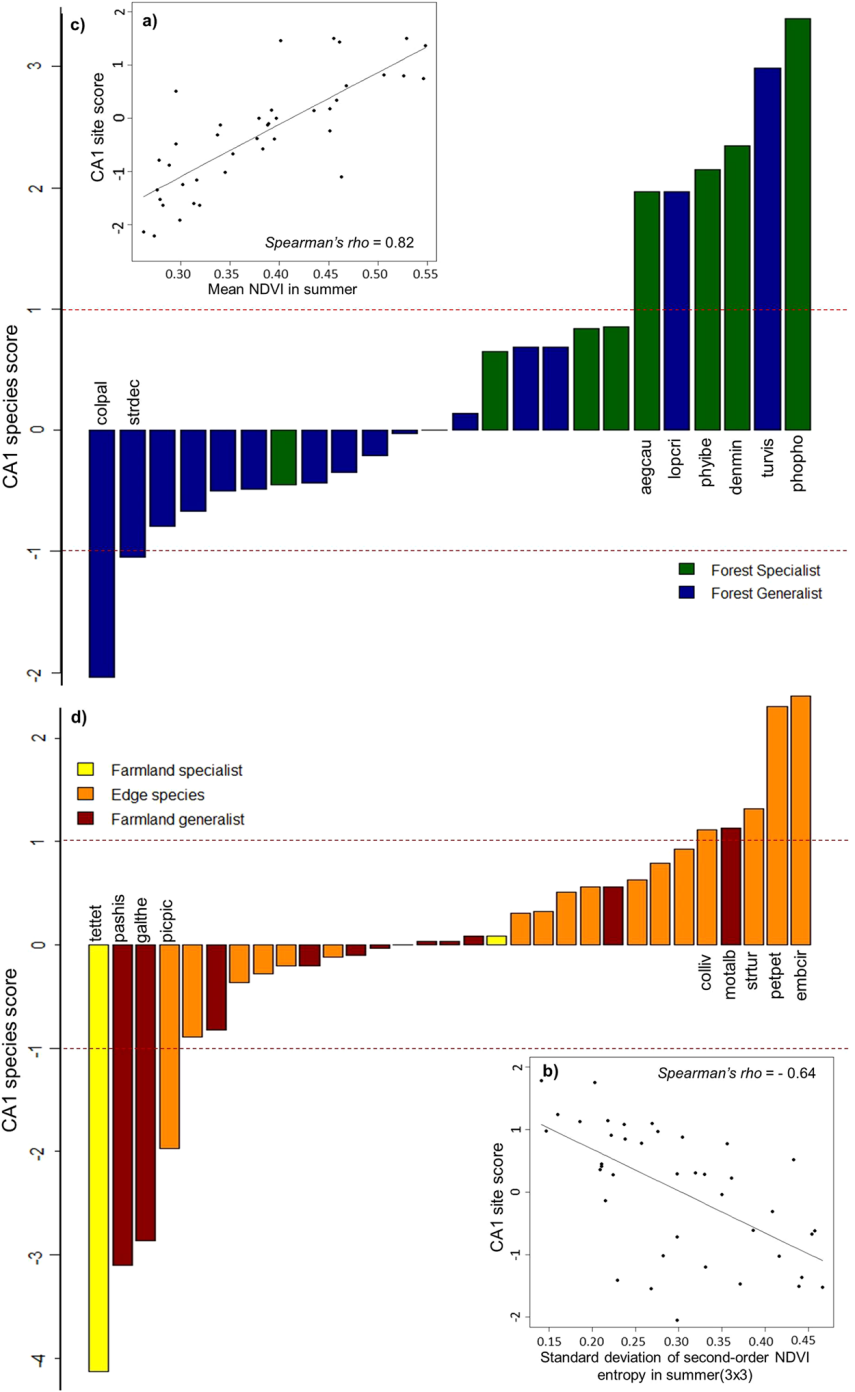
Relationship between site scores in the first principal axis (CA1) of the correspondence analysis and the environmental variables retained by the GDMs for forest bird species (**a**) and open-land bird species (**b**). Species scores (**c**,**d**) in the first principal axis (CA1) of the correspondence analysis. Species with a score > 1 are identified. Species full name is presented in Supplementary Table S3.

Site scores in the first principal axis (CA1) of correspondence analyses conducted for forest species and open-land species were correlated with the NDVI_mn_SU (Spearman’s rho = 0.82) for forest species (Fig. 3a), and with the NDVI_ent3x3_sd_SU (Spearman’s rho = −0.64) for open-land species (Fig. 3b). Forest specialist species, such as *Phoenicurus phoenicurus*, *Aegithalos caudatus* or *Dendrocopus minor*, had high CA1 scores thus being more related to landscapes with high NDVI_mn_SU values (Fig. 3c). For open-land species, the analysis suggests two groups: farmland species, such as *Tetrax tetrax*, more associated to higher values of NDVI_ent3x3_sd_SU, and edge species, such as *Emberiza cirlus* and *Petronia petronia*, more associated to lower values (Fig. 3d).

## Discussion

We used open-access satellite imagery and biodiversity data to investigate the relationship between the richness and dissimilarity of forest and open-land bird communities and variables of habitat extent and structure. We found that NDVI texture variables, with a 30m-pixel resolution, were in general good indicators of species richness at the landscape level, performing better than land cover variables of the extent of the main habitat. Our results also suggest that the availability of tree cover in the landscape is more important for forest birds than the extent or structure of the forest habitats present in the landscape, and that open-land birds respond both to the structure of their main habitat and to the overall structure of the landscape. Moreover, structure variables were more important to explain community dissimilarity patterns of the two species groups in the study region than climate variables or geographic distance. Finally, we tested the use of satellite imagery collected in spring and summer, as the images from different seasons capture different landscape elements according to the phenological stage. We found that summer images are particularly useful because they capture the distribution of the green perennial vegetation, to which both forest and open-land birds respond. Moreover, spring images appear to be relevant for open-land birds to monitor vegetation within open-land habitats.

More specifically, forest birds’ richness was best described by models that included the mean NDVI in summer (NDVI_mn_SU) and the range of solar radiation in the landscape. In Mediterranean systems, NDVI_mn_SU mostly captures the vegetation that stays green in the dry summer season, that is, perennial trees and shrubs^30^. NDVI_mn_SU was strongly correlated to forest birds’ richness, but only moderately correlated to the proportion of oak forest and to the area of the largest oak forest patch in the landscape. Moreover, forest bird richness was only moderately correlated to these land cover variables (Table 1). Hence, NDVI_mn_SU probably provides a more accurate measure of the tree cover in the landscape, and therefore of habitat availability for forest birds, being a better descriptor of forest bird richness. Wood *et al*.^15^ also show that mean NDVI is a good indicator of vegetation structure among habitats, because it captures the transitions in the landscape, especially if the habitats clearly differ in terms of structure, such as in grassland – woodland mosaics.

The strong association between forest bird richness and the NDVI_mn_SU could also be related to ecosystem productivity, as areas of high plant productivity would be associated to a higher availability of foraging resources^22^. However, even if this is the case in our study, the results are not explicit enough to confirm it. First, we did not find a similar association for open-land bird richness or for the total species richness. Second, the correlation of species richness with the mean NDVI in spring (NDVI_mn_SP), which represents vegetation productivity at its peak, was not only weak but also negative for all the species groups, possibly because this variable is capturing the signal of both forest and open-land habitats. Regarding solar radiation range, this variable is related to the complexity of landscape morphology, characterized by parameters such as slope, aspect, and surface roughness. Hence, our results suggest that forest birds are more associated to landscapes with a more complex morphology, with diverse slopes and solar exposition that create the microclimate favorable to the growth of oak trees^31,32^.

Contrary to our expectation, our results suggest that the availability of tree cover in the landscape, indicated by NDVI_mn_SU and by LgtPtch_OF, may exert a stronger influence on forest bird richness than the within-habitat structure of oak forest patches (e.g., tree density, tree cover arrangement). A similar result, also for Alentejo, was reported by Santana *et al*.^33^; the authors found a strong association of woodland bird species richness to the amount of woodland in the landscape but not to landscape configuration. This finding is further supported by the GDM results, which suggest that the composition of forest bird communities tends to stabilize, with limited addition of new species or species replacement, above a threshold value of the NDVI_mn_SU (Fig. 2a).

The lack of response from forest birds to within-habitat structure may also be related to data limitations, since previous studies have reported such effect. For instance, grazing management and changes in tree density have been reported to affect bird communities in wood-pastures^34,35^. In our study, bird data were aggregated for the landscape mosaic and the satellite data probably misses most or part of the signal of the forest understory, even if understory features are correlated to canopy features or affect the reflectance of the canopy layer^15,24^. Moreover, the selection of the NDVI_mn_SU by both the GLM and the GDM analyses, and the partition of community similarity (Supplementary Fig. S2a), suggest that differences in species richness are an important component of community dissimilarity in the study area. This is could be due to the resolution of the bird data, as discussed above, but also to the extent of the study area. That is, species replacement will be moderated when communities within landscapes with identical forest cover are compared at the scale of the Alentejo region. Moreover, the effect of geographic distance on community dissimilarity was found to be smaller than the effect of landscape structure for forest birds (Fig. 2b). On the other hand, the species found to be more associated to high NDVI_mn_SU values were forest specialists (Fig. 3c), such as *Phoenicurus phoenicurus*, *Aegithalos caudatus* and *Dendrocopos minor*, suggesting that these species preferentially occur in landscapes where NDVI_mn_SU, an indicator of tree cover, reaches higher values.

Open-land bird richness was best described by models that included second-order texture variables related to the arrangement of the vegetation in the landscape in general (NDVI_ent3x3_sd_SU, NDVI_mn_SU) and in the open-land habitats (NDVI_var9x9_sd_OP_SP, NDVI_ent9x9_mn_OP_SP, NDVI_var3x3_mn_OP_SU). At the landscape scale, NDVI_ent3x3_sd_SU was moderately correlated to open-land bird richness (Table 1) and was collinear to the proportion of open-land habitats and to the area of the largest patch of open-land. Higher values of NDVI_ent3x3_sd_SU correspond to coarse-grained landscapes composed of large patches of homogenous land with sharp transitions between them (i.e., high variation of the NDVI entropy measured in moving windows within the landscape), for instance, where large patches of crop land are delimited by edge habitats. Regarding NDVI_mn_SP, this variable is associated to landscapes with high oak forest cover, just like NDVI_mn_SU, and was negatively correlated with open-land bird richness (Table 1). All the variables selected at the habitat level, NDVI_var9x9_sd_OP_SP, NDVI_ent9x9_mn_OP_SP, and NDVI_var3x3_mn_OP_SU were weakly correlated to open-land bird species richness (Table 1). Therefore, their indicator role should be interpreted with caution, especially the last two last that have low relative importance (Table 2). As regards NDVI_var9x9_sd_OP_SP this variable is relatively important (Table 2) and therefore, a potential indicator of open-land bird richness (Table 2), with higher values of richness being associated to homogenous open-land patches (i.e., similar values of NDVI variance in moving windows).

As observed for forest birds, the dissimilarity between open-land bird communities was mostly explained by the gradient of a key descriptor of species richness, here NDVI_ent3x3_sd_SU (Fig. 2). Also, community composition appears to stabilize above a threshold value. In this case, the partition of community dissimilarity (Supplementary Fig. S2b) suggests that species replacement is an important component of community dissimilarity, which, as discussed next, could be associated to a gradient between different types of open-land habitats. Nevertheless, the deviance explained by the model and the relative importance of NDVI_ent3x3_sd_SU are relatively low (Table 3), leaving a large share of compositional dissimilarity unexplained. As found in previous studies, the composition of open-land bird communities is very responsive to management and to the composition of crops^33,36,37^, factors that were not tested in this study but likely to be relevant. Another important aspect regards the structure of the species group, which encompasses both farmland and edge species that have different ecological preferences within the open-land habitats. This may prevent the detection of a strong response pattern for the group as a whole. In fact, farmland species appear to be more associated to landscapes with high values of NDVI_ent3x3_sd_SU (Fig. 3d), which are also richer in species, and edge species to finer-grained landscapes, with higher edge density and less sharp transitions, that have lower values of NDVI_ent3x3_sd_SU (Fig. 3d).

The best models for total species richness were less effective in explaining richness patterns than the models for the species groups (Table 2), possibly because forest and open-land birds respond differently to landscape attributes. Overall, the positive association with the radiation range and with NDVI_var3x3_mn_SU suggests that total species richness is higher in landscapes with high heterogeneity at the landscape and the local scale (i.e., 3 × 3 pixel windows). This result agrees with previous studies that reported the effect of habitat heterogeneity in species richness^3,22,38^. The remaining variables included in the best models have low relative importance and therefore their indicator value is less clear and of marginal importance to explain total species richness. In contrast to species richness patterns, community dissimilarity was well described by the GDM for all species (Table 3). The turnover in community composition is distinct and well explained by a land cover gradient between forest and open-land habitats (note that the two variables are inversely correlated in our sample), with species replacement slowing down (i.e., flatter slope) at intermediate levels of the gradient (Fig. 2). NDVI_var3x3_mn_SU and NDVI_sd_SU were also identified as significant descriptors of community dissimilarity. The effect of these texture variables is probably related to compositional changes driven by landscape heterogeneity. Also, both are summer variables which highlight the importance of perennial vegetation in shaping landscape structure and providing habitat diversity for bird communities.

Our results agree with the findings from previous studies that demonstrate the potential of open-access data to be used in ecological research and biodiversity monitoring, and inform land management and decision-making, especially when financial resources are limited^39–41^. However, there were limitations that restricted the data potential to describe species response to vegetation structure. First, although NDVI texture variables appear to be good indicators of vegetation horizontal structure, the use of optical imagery is not adequate to capture vertical structure, especially in multilayered systems, notably forests. The use of data from active sensors, such as the Synthetic Aperture Radar (SAR) and the Light Detection and Ranging (LiDAR), could help to address this limitation and produce enhanced spatial layers of habitat structure^14,16,24^. However, while promising, current data availability from active sensors is limited, and data processing and interpretation still require advanced technical skills that constrain their use^16,17^. Moreover, while spring images appeared to be relevant to monitor vegetation within open-land habitats, the adequacy of the data at the landscape scale is affected by the simultaneous reflectance, at peak productivity, of the herbaceous and the woody components. The extraction of the herbaceous component from spring images could provide a solution to work around this limitation^30^.

Second, regarding biodiversity data, the quality of data from open-access data repositories is affected by several problems, namely the uneven sampling effort, in space and time, and biases related to species detectability (i.e., conspicuous species are reported more commonly)^39,40^. To address these issues we performed a multistep protocol to ensure data quality prior to data analyses (the steps are described in detail in the Methods and summarized in Supplementary Fig. S1). Briefly, we restricted our species pool to resident birds, defining a priori the species to include in the dataset, we aggregated data spatially (i.e., landscape-sized cells) and temporally (i.e., time windows) to enhance spatial accuracy and taxonomic coverage, we used species richness estimators to check inventory completeness per cell and selected only the well-surveyed cells and we defined land cover rules to avoid confounding effects from land cover types other than oak forest and open-land habitats. After implementing these filters, only 20% of the available records, retrieved from GBIF, were kept in the final dataset. Moreover, the cost of enhancing data spatial accuracy was losing spatial resolution, which, as already discussed, may have impaired the detection of species responses to vegetation structure at the finer scales. Unsolved issues include the uneven coverage of the study area and potential errors in species identification by data providers, which would be difficult to detect since the distribution range of most species, in the selected species pool, covered the study area.

Systematic field sampling, as counterpoint to open-access data, is not affected by the above data limitations and provides more reliable data, yet it is constrained by the costs involved, especially if a high sampling effort is required at a large spatial extent. In those cases, open-access data may provide a cost-effective alternative. At the same time, it is necessary to promote data sharing, reward data providers and establish data quality standards to enhance the availability and the quality of data from public databases^18^.

This study illustrates the potential of open-access biodiversity data and satellite imagery as cost-effective data sources to address trade-offs between sampling extent and sampling effort and to support biodiversity monitoring. In particular, our findings suggest that NDVI texture variables can be used to monitor the effects of changes in vegetation structure on bird communities.

## Methods

### Study area

Our study area is the Alentejo region (NUTS II) in Portugal (Fig. 1). This is a predominantly rural region with low population density. The climate is Mediterranean with hot and dry summers^42^. The landscape has a gentle topography with extensive land uses, but intensification is increasing with potential impacts on biodiversity^43–46^. Most notably, montados (*dehesas* in Spanish) cover a significant share of the landscape. These are traditional systems with a silvo-pastoral use, where cork oak (*Quercus suber*) and holm oak (*Q*. *rotundifolia*) are the dominant trees, forming pure or mix stands with a tree cover varying between 10% and 30%^47^. Montados are managed for multiple productive purposes, the most important being cork extraction, pastures and livestock^48,49^. The multifunctional use promotes habitat structural diversity, which combined with the large regional extent and the generally low human population density enables the persistence of many species, including endangered species^38,49^. For the purpose of this study, all forest stands of the Portuguese land cover map, COS2007*v2*.0^47^ (http://www.dgterritorio.pt/) that are dominated by oaks (≥75%), and with a tree density of at least 10% were designated as oak forest. For data analysis, we used a level 5 *geohash* grid (www.geohash.org), to divide the study area in landscape-level cells of approximately 4.89 km × 4.89 km (Fig. 1, see Supplementary Fig. S1).

### Study design

To account for differences in habitat use by forest and open-land species, we assigned a distinct set of candidate variables to each species group (Supplementary Table S1), including NDVI texture variables, measured in spring or in summer, at the landscape (i.e., using all pixels in the grid cell) or at the habitat scale (i.e., using only the pixels overlapping the preferred habitat, either forest or open-land habitats, in the Portuguese land cover map layer). The spring imagery is expected to provide a better signal of the vegetation cover of open-land habitats, when the herbaceous vegetation is at peak productivity, while the summer imagery, will capture the contrast between the senescent herbaceous cover and the perennial vegetation, thus providing better information on tree cover. Regarding the two scales, the landscape and the habitat scale, the aim is to account for species’ responses to the overall surrounding landscape and to the preferred habitat. In addition to NDVI measures, all sets of candidate variables included other environmental variables, measured at the landscape scale, namely climatic, topographic and land cover variables. From the initial list of 70 candidate variables (see Supplementary Table S1), we retained a final set of non-collinear variables per species group (Table 1). Variable selection is described in the section *Data analysis*.

### Climate, elevation and land cover data

Precipitation, temperature, and solar radiation data were collected from WorldClim database (http://www.worldclim.org/)^50^ on a 1 km resolution, elevation data were obtained from the *Digital Terrain Model* (30 m × 30 m) for Portugal in ArcGis *Online* 10.3.1 (https://www.arcgis.com/) with an accuracy of 7–14 m. Land cover data were extracted from the Portuguese land cover map, COS2007*v2*.0 (minimum mapping unit 1 ha). For this study, we aggregated land cover classes into five categories: open land (permanent and temporary pastures, sand dunes, vineyards, shrubs and sparse vegetation), oak forest (open or closed forests and agro-forestry systems dominated by oaks), other forests (open and closed forests dominated by species other than oaks), urban (all the areas described at COS2007*v2*.0 -level 1 as artificialized territory, including industries and roads), and water bodies (all the areas described at COS2007*v2*.0 - level 1 as water bodies). To better capture species response to oak forest systems and reduce the influence of other forest types in the landscape, we defined eligible cells as those with a maximum of 20% cover of other forest types and where the cover of oak forest was not smaller than the cover of other forests (i.e., max 20% cover of other forest and oak forest cover ≥ other forest cover). The final sample included 59 cells.

### Remote sensing data

Six Landsat-5 images, free of clouds, corresponding to 20^th^ March 2011 and 20^th^ July 2011 (path 203 and rows 33 and 34); 25^th^ April 2010 and 18^th^ August 2011 (path 204 and row 33), were used. These images were downloaded from the United States Geological Survey (USGS) Earth Explorer server, selecting the Landsat Surface Reflectance Level-2 Science Products (https://landsat.usgs.gov/landsat-surface-reflectance-data-products). Surface Reflectance products (at 30-meter spatial resolution) provide an estimate of the surface spectral reflectance as it would be measured at ground level in the absence of atmospheric scattering or absorption^51^. In order to monitor vegetation structure, the NDVI was computed. This index is based on the normalized ratio between absorbed red light and reflected near infrared light^52^. NDVI values range from −1 (non-photosynthetically active vegetation) to +1 (highly photosynthetically active vegetation). This index has been used successfully in several studies to evaluate land cover performance^53^ or phenological information^54^ or plant-community degradation^55^ or to supply information about crops^56^. Urban and water areas (NDVI ≤ 0) were masked from the original NDVI images to remove their signal from image texture analysis.

Image texture measurements quantify the spatial variation and arrangement of the reflectance values of neighboring pixels, expressing the level of spectral heterogeneity in a given area^57^. Three first-order texture variables (NDVI entropy, NDVI mean, NDVI standard deviation) and two second-order texture variables (NDVI entropy, NDVI variance) were calculated for each cell and for the main habitat subsets, as listed in Supplementary Table S1.

First-order texture variables do not consider pixel neighbor relationships and are measured using the original image values within a certain group of pixels^57^, which in our case was the full cell. On the other hand, second-order variables consider the spatial relation between neighboring pixels within a moving window^22^, using a gray-level co-occurrence matrix (GLCM), which contains the probabilities of co-occurrence of values for pairs of pixels^58^. The *GLCM R* package was used to extract second-order texture measures within a 3 × 3 and a 9 × 9 moving window in four directions (0°, 45°, 90°, 135°). The *Zonal Statistics Tool* from ArcGIS 10.3.1 was used to summarize the mean and standard deviation and obtain a single texture value for each cell.

### Bird data

Georeferenced bird occurrence data was retrieved from the digital database of GBIF (Global Biodiversity Information Facility, http://www.gbif.org/)^59^. We searched for species with resident populations in the study area and that use open-land and oak forest habitats (Supplementary Table S3). The taxonomic nomenclature and the species distribution were retrieved from Catry *et al*.^60^. We excluded from our list exotic species, species with wide territories, such as eagles and hawks, species with nocturnal habits, such as owls and nightjars, and insectivorous aerial birds, such as swallows and swifts^61^.

For the selected species, we collected species occurrences dated from April to June, to cover the birds’ nesting and reproduction periods^27^, between 2005 and 2015. Only records with no geospatial issues and under the *CCO* 1.0 use license were used. The retrieved bird occurrence dataset, for the total number of searched years (i.e., 2005–2015), consisted of 122110 records (dataset available at, 10.15468/dl.pwrz9h).

### Selection of well-surveyed cells

Following this, an identification of the cells for which species occurrences provided adequate inventories^62,63^ was performed. For this purpose, data were analysed for each individual year, and aggregated in 2-year and 3-year time windows to increase sample size and taxonomic coverage. The following steps were repeated for all possible 1-year, 2-year and 3-year contiguous time windows to identify well-surveyed cells. First, cells with less than 20 observed species were excluded^64^. Second, two complementary methods were applied to estimate total cell species richness: non-parametric estimators based on the number of rare species (Chao 2 and Jackknife 1)^10^, and the number of estimated species at the 95% upper confidence interval of the accumulation curve produced with the Mao Tau analytical function^65^. The three estimates were obtained with EstimateS 9.1.0^66^. Based on the assumption that a higher number of records in a grid cell represents a higher survey effort, the total number of occurrence records in each cell was used as a surrogate of sampling effort^67^.

Inventory completeness was calculated by relating the maximum estimated species richness among the three estimators (i.e., Mao Tau, Chao 2 and Jackknife 1) and the observed richness, that is, observed/maximum estimate × 100. Only the cells with completeness equal to or greater than 75% were considered as well-surveyed^62^. From a total of 1839 grid cells in the study area (Fig. 1), 1060 cells had GBIF records for the selected bird species. The number of cells suited for analysis decreased sharply after assessing inventory completeness. The best results (i.e., higher number of well-surveyed cells) were found for the 3-year time windows: 94 cells in 2007–2009, 91 cells in 2010–2012 and 68 cells in 2013–2015. By intersecting the 59 cells with adequate environmental data (see *Climate*, *elevation and land cover data* section) with the cells with well-surveyed bird data, we were able to match a maximum of 41 cells for the 2010–2012 time window (Fig. 1, see Supplementary Fig. S1 and Supplementary Table S4). Finally, after fitting generalized linear models (see section *Data analysis*), an over influential cell that had a Cook’s distance larger than 1 was detected. This cell was removed from the sample, resulting in a final sample of 40 cells (7858 records) that were used in data analyses.

### Species groups

The information in Pereira *et al*.^27^ was used to divide the 78 species present in our sample of 40 cells into two species groups: 27 forest bird species (10 specialists and 17 generalists) and 51 open-land bird species (8 farmland specialists, 12 farmland generalists, 22 edge species and 9 species requiring special landscape elements associated to farmland).

### Data analysis

From each set of candidate variables per species group, we selected a group of non-collinear variables to be used in the statistical models (Table 1). First, we calculated the Spearman’s pairwise correlations among all pairs of candidate variables, and among candidate variables and the response variables (i.e., the observed species richness of each species group and all species (Supplementary Table S5)). The candidate variables that were weakly correlated with the response variable (−0.1 < Spearman’s rho < 0.1) were removed^68^. For the remaining candidate variables, if two or more variables were strongly correlated (Spearman’s rho > |0.7|), we only kept the one most correlated with the response variable^68,69^. Finally, we checked that the variation inflation factors (*vif* function in R-package *car*) of the remaining variables was lower than 5. We also tested for the presence of spatial autocorrelation in the response variables using *Moran’s I statistic* (*dnearneigh*, *nb2listw*, and *moran*.*test* functions in R-package *spdep*). None of the response variables was spatially auto-correlated.

Generalized linear models (GLM), with *Poisson* error distribution and *log link* function, were used to assess the importance of the environmental variables in shaping species richness patterns. We used GLMs because our response variables (count data) were fit to a Poisson distribution. The variables listed in Table 1 were also tested for quadratic relationships. For each candidate variable, we compared the corrected Akaike Information Criterion (AICc) of the regression model comprising only the linear term of the variable against the response variable, or the linear plus the quadratic term. If the regression model with the quadratic term had a better fit, both the linear and the quadratic term were included in the full GLM with the final set of candidate variables. Quadratic terms were retained for mean elevation for all species and for percentage of water areas for the forest species group.

We used the R-package *glmulti*^70^ to test all possible combinations of the variables listed in Table 1 per species group (i.e., full model) and rank the best models using AICc. Best GLMs (difference from AICc_*minimum*_ < 2)^71^ were used to identify the most important variables affecting species richness patterns. Because we had a small sample size of 40 cells we only tested for main effects. If a quadratic term was retained in one of the best models, the linear term was forced in the model^72^. We selected the most parsimonious model with the lowest AICc to check for overdispersion (*dispersiontest* function in R-package *AER*); no evidence of overdispersion was found. The relative importance of each candidate variable was estimated by summing the Akaike model weights over all models in the confidence set^71^. The model-averaged parameter estimates, the unconditional standard errors and the 95% confidence intervals (*coef*.*glmulti* function in R-package *glmulti*^70^) are presented in Supplementary Table S2.

To identify the variables driving species turnover in bird communities, we applied generalized dissimilarity modelling using the R-package *gdm*^73^. GDM uses generalized linear modeling to accommodate the curvilinear relationship of both non-linear distance relationship and non-constant rate of turnover along gradients^74^. This regression allows dissimilarities to be estimated for all pairs of sites (grid cells) and the inclusion of environmental data. Also it makes the reasonable assumption that compositional dissimilarity can only increase with increasing separation of sites along the environmental gradient^75,76^.

First, to fit GDMs, we used the final set of non-collinear variables to create a GDM site-pair matrix for each species group. In addition to the environmental variables, geographical coordinates of cell centroids were included in the matrix to compute geographical distance. Variable significance was tested by combining Monte Carlo sampling and stepwise backward elimination as executed in the *gdm*.*varImp* function, with 250 permutations per step until only significant (α < 0.05) variables remained in the model. Second, to assess the amount of variance explained by each variable and the model, we fitted GDMs using *gdm* function, using only the significant variables obtained with the *gdm*.*varImp* function. We used the default of three *I-spline* basis functions per variable. We summed the coefficients of the *I-splines*, which are partial regression fits, to assess the relative importance of each variable in describing patterns of beta diversity^77^. We also plotted the *I-splines* to visualize the variation and magnitude of species turnover along gradients of the significant variables. The maximum height obtained by the curve represents the total amount of compositional turnover associated with that variable, holding all other variables constant. The slope of the *I-spline* indicates the rate of species turnover^77^. To better understand the processes underlying compositional changes, we used the *beta*.*pair* function in the R-package *betapart*^78^ to decompose the overall community dissimilarity in the two additive components of species replacement and difference in species richness^79^.

To further test the explanatory role of the environmental variables found to have a significant effect on compositional dissimilarity, a correspondence analysis was applied to the matrix of species presence per site (i.e., cell), and the scores of the sites in the first principal axis were correlated against the environmental variables to test their association with species communities. Then, species scores were used to identify the species more related to the communities at the ends of the environmental gradient (i.e., with extreme scores). Only the species observed in at least 5 cells were included in the correspondence analyses, which excluded three of the 27 forest species and 20 of the 51 open-land species.

## Supplementary information

Supplementary Information

## Data Availability

The datasets generated and analyzed during the current study are available in the GBIF.org repository, at 10.15468/dl.pwrz9h. The remaining data generated or analyzed during this study are included in this published article (and its Supplementary Information files).
