# Supplementary material for: Remotely sensed indicators and open-access biodiversity data to assess bird diversity patterns in Mediterranean rural landscapes
Source: Sci Rep. 2019 May 2;9:6826. doi: 10.1038/s41598-019-43330-3 (PMC6497664; doi:10.1038/s41598-019-43330-3)
Supplement: Supplementary file 1 — Supplementary Information [file 41598_2019_43330_MOESM1_ESM.pdf]

Suplementary information for:

Remotely sensed indicators and open-access biodiversity data to assess bird diversity patterns in Mediterranean rural landscapes.

Inês Ribeiro[a] , Vânia Proença[a],*, Pere Serra Ruiz[b] , Jorge Palma[a] , Cristina Domingo-Marimon[c] , Xavier Pons[b] , Tiago Domingos[a]

[a] MARETEC, Instituto Superior Técnico, Universidade de Lisboa, Av. Rovisco Pais 1, 1049-001 Lisboa, Portugal

[b] Grumets Research Group, Department of Geography, Universitat Autònoma de Barcelona, 10 Edifici B, Campus de la UaB, Barcelona, Spain

[c] Grumets Research Group, CREAF, Campus UAB, Edifici C, Cerdanyola del Vallès, Catalonia, 08193, Spain

* Corresponding author: vania.proenca@tecnico.ulisboa.pt

Contents:

Figure S1. Summary of data filtering steps to collate the final dataset of 40 cells suitable for analysis.

Table S1. Full list of candidate predictor variables per species group.

Figure S2. Partition of community dissimilarity

Table S2. Model-averaged parameter estimates, unconditional standard errors, importance, 95% CI

Table S3. Full list of observed species, species habitat preference, and number of cells in which they were present.

Table S4. Full list of well-surveyed cells in the 2010-2012 time window.

Table S5. Observed richness of species groups in the 40 cells selected for data analyses.

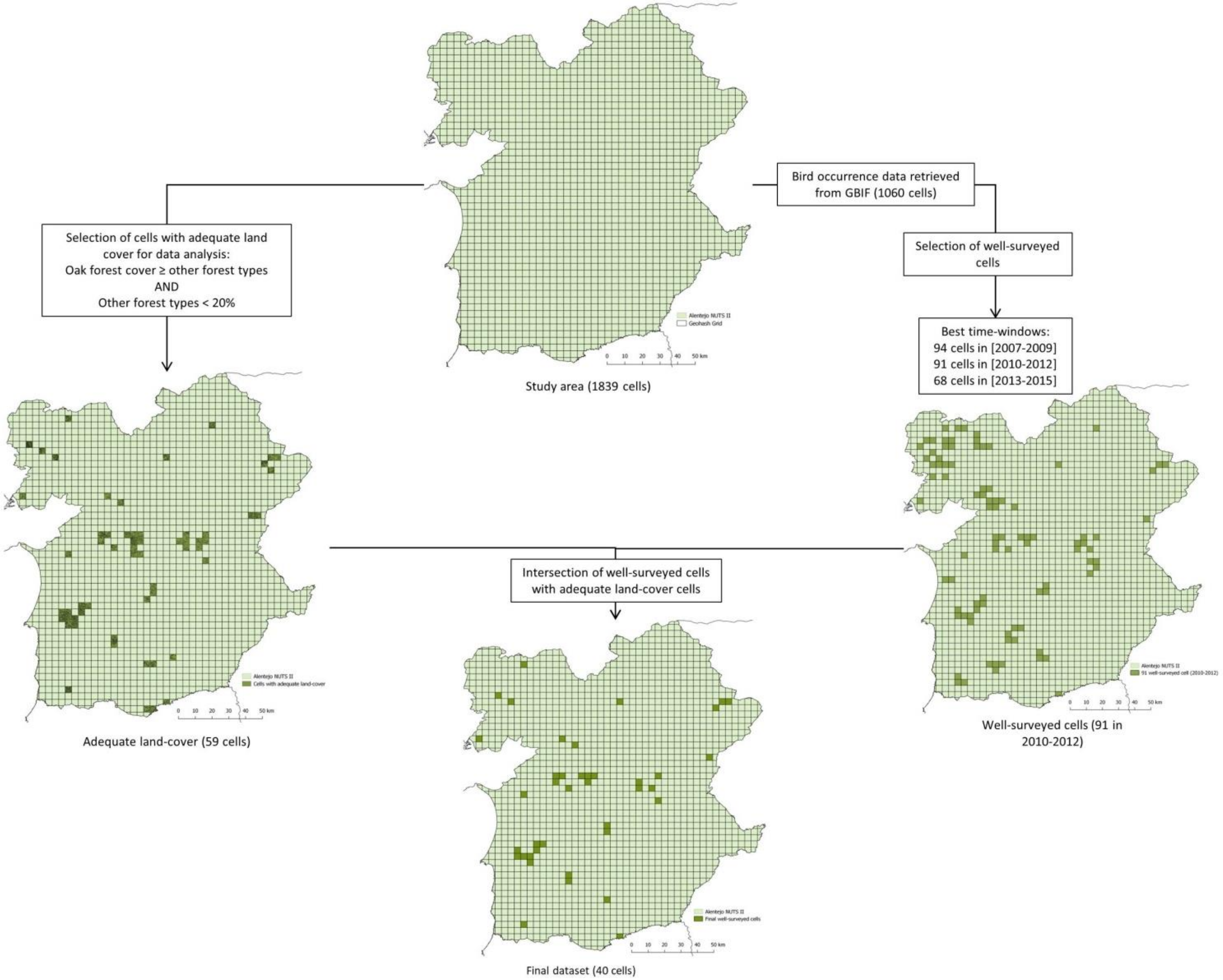

Supplementary Figure S1. Summary of data filtering steps to collate the final dataset of 40 cells suitable for analysis.

Note: The intersection of the cells with adequate land cover with the best time windows resulted in the maximum number of 41 cells in the 2010-2012 time window. However, after fitting generalized linear models (see section Data analysis) an over influential cell with a Cook's distance larger than 1 was detected. This cell was removed from the sample, resulting in the final sample of 40 cells (7858 records).

Supplementary Table S1. Full list of candidate predictor variables (shaded cells) and final set of non-colinear variables retained after variable selection (marked with "X") per species group. Variables are organized by main category (climate, topographic, land cover and NDVI texture) and scale (landscape and habitat scale). Landscape scale refers to variables measured for the full grid cell (i.e., using all pixels); habitat scale refers to variables measured using only the pixels overlapping patches of the preferred habitat, either forest or open-land habitats. Main habitats are the oak forest for forest bird species and open habitats for open-land bird species. NDVI texture variables were measured for the full cell (first-order variables) and using 3 x 3 and 9 x 9 pixel windows (second-order variables).

Landscape scale	*Variable code*	*All Species*	*Forest Birds*	*Open-land Birds*
**Climate variables**				
Mean Annual Precipitation (mm)	AnPrecip_mn	X		
Mean Trimestral Temperature (Celsius)	TmeanT			X
Minimum Temperature in April (Celsius)	TminA		X	
Maximum Temperature in June (Celsius)	TmaxJ	X		
Mean Solar Radiation	MnRad			
Solar Radiation Range	RadRg	X	X	
**Topographic variables**				
Mean Elevation (meters)	Elev_mn	X		
**Land Cover variables**				
Percentage cover of Urban area	%UrbnAr	X	X	X
Percentage cover of Open land	%OpnAr	X		
Percentage cover of Oak Forest	%OakFor			
Percentage cover of Other Forest	%OthFor	X	X	
Percentage cover of Water areas	%WterAr		X	X
**Texture variables**				
Full cell – 1st order variables				
Mean Value of NDVI in Summer	NDVI_mn_SU		X	
Standard deviation of NDVI in Summer	NDVI_sd_SU	X		
NDVI entropy in Summer	NDVI_ent_SU			
Mean Value of NDVI in Spring	NDVI_mn_SP	X	X	X
Standard deviation of NDVI in Spring	NDVI_sd_SP	X		X
NDVI entropy in Spring	NDVI_ent_SP			
3x3 moving window – 2nd order variables				
Mean of NDVI entropy in Summer	NDVI_ent3x3_mn_SU			
Standard deviation of NDVI entropy in Summer	NDVI_ent3x3_sd_SU			X

Mean of NDVI entropy in Spring	NDVI_ent3x3_mn_SP			
Standard deviation of NDVI entropy in Spring	NDVI_ent3x3_sd_SP		X	
Mean of NDVI variance in summer	NDVI_var3x3_mn_SU	X		
Standard deviation of NDVI variance in Summer	NDVI_var3x3_sd_SU			
Mean of NDVI variance in spring	NDVI_var3x3_mn_SP			
Standard deviation of NDVI variance in Spring	NDVI_var3x3_sd_SP			
9x9 moving window – 2nd order variables				
Mean of NDVI entropy in Summer	NDVI_ent9x9_mn_SU			
Standard deviation of NDVI entropy in Summer	NDVI_ent9x9_sd_SU			
Mean of NDVI entropy in Spring	NDVI_ent9x9_mn_SP	X		
Standard deviation of NDVI entropy in Spring	NDVI_ent9x9_sd_SP			
Mean of NDVI variance in summer	NDVI_var9x9_mn_SU			
Standard deviation of NDVI variance in Summer	NDVI_var9x9_sd_SU			
Mean of NDVI variance in spring	NDVI_var9x9_mn_SP			
Standard deviation of NDVI variance in Spring	NDVI_var9x9_sd_SP		X	
Habitat scale				
**Largest patch of main habitat**				
Area of the Largest Patch of Oak Forest (km2)	LgtPtch_OF		X	
Area of the Largest Patch of Open land (km2)	LgtPtch_OP			
**Texture variables**				
3x3 moving window – 2nd order variables				
Mean of NDVI entropy in Oak forests in Summer	NDVI_ent3x3_mn_OF_SU		X	
Standard deviation of NDVI entropy in Oak forests in Summer	NDVI_ent3x3_sd_OF_SU			
Mean of NDVI entropy in Oak forests in Spring	NDVI_ent3x3_mn_OF_SP			
Standard deviation of NDVI entropy in Oak forests in Spring	NDVI_ent3x3_sd_OF_SP			
Mean of NDVI variance in Oak forests in Summer	NDVI_var3x3_mn_OF_SU			
Standard deviation of NDVI variance in Oak forests in Summer	NDVI_var3x3_sd_OF_SU			
Mean of NDVI variance in Oak forests in Spring	NDVI_var3x3_mn_OF_SP			
Standard deviation of NDVI variance in Oak forests in Spring	NDVI_var3x3_sd_OF_SP		X	
Mean of NDVI entropy in Open land in Summer	NDVI_ent3x3_mn_OP_SU			
Standard deviation of NDVI entropy in Open land in Summer	NDVI_ent3x3_sd_OP_SU			
Mean of NDVI entropy in Open land in Spring	NDVI_ent3x3_mn_OP_SP			
Standard deviation of NDVI entropy in Open land in Spring	NDVI_ent3x3_sd_OP_SP			
Mean of NDVI variance in Open land in Summer	NDVI_var3x3_mn_OP_SU			X

Standard deviation of NDVI variance in Open land in Summer	NDVI_var3x3_sd_OP_SU		
Mean of NDVI variance in Open land in Spring	NDVI_var3x3_mn_OP_SP		
Standard deviation of NDVI variance in Open land in Spring	NDVI_var3x3_sd_OP_SP		
9x9 moving window – 2nd order variables			
Mean of NDVI entropy in Oak forests in Summer	NDVI_ent9x9_mn_OF_SU		
Standard deviation of NDVI entropy in Oak forests in Summer	NDVI_ent9x9_sd_OF_SU		
Mean of NDVI entropy in Oak forests in Spring	NDVI_ent9x9_mn_OF_SP		
Standard deviation of NDVI entropy in Oak forests in Spring	NDVI_ent9x9_sd_OF_SP	X	
Mean of NDVI variance in Oak forests in Summer	NDVI_var9x9_mn_OF_SU		
Standard deviation of NDVI variance in Oak forests in Summer	NDVI_var9x9_sd_OF_SU		
Mean of NDVI variance in Oak forests in Spring	NDVI_var9x9_mn_OF_SP		
Standard deviation of NDVI variance in Oak forests in Spring	NDVI_var9x9_sd_OF_SP		
Mean of NDVI entropy in Open land in Summer	NDVI_ent9x9_mn_OP_SU		
Standard deviation of NDVI entropy in Open land in Summer	NDVI_ent9x9_sd_OP_SU		
Mean of NDVI entropy in Open land in Spring	NDVI_ent9x9_mn_OP_SP		X
Standard deviation of NDVI entropy in Open land in Spring	NDVI_ent9x9_sd_OP_SP		
Mean of NDVI variance in Open land in Summer	NDVI_var9x9_mn_OP_SU		
Standard deviation of NDVI variance in Open land in Summer	NDVI_var9x9_sd_OP_SU		
Mean of NDVI variance in Open land in Spring	NDVI_var9x9_mn_OP_SP		X
Standard deviation of NDVI variance in Open land in Spring	NDVI_var9x9_sd_OP_SP		X

Figure S2. **P**air-wise community dissimilarities and geographic distance for forest birds (a), open-land birds (b) and all species (c). Total dissimilarity (βsor, open circles) is decomposed in the two additive components of species replacement (βsim, blue circles) and differences in species richness (βsne, red circles). Total dissimilarity (βsor) = βsim + βsne, for each pair of sites.

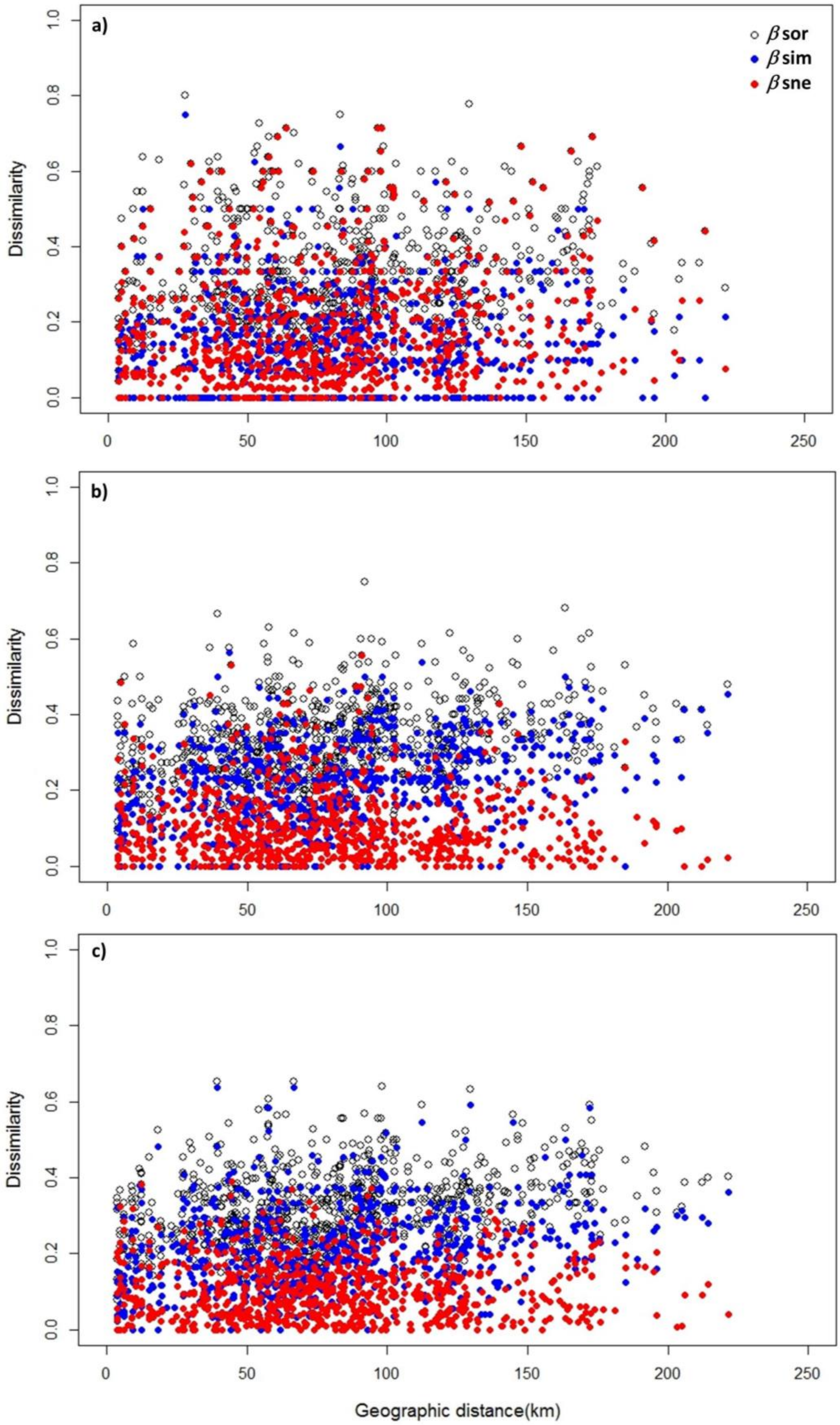

Supplementary Table S2. Model-averaged parameter estimates, unconditional standard errors, importance, and 95% confidence intervals in the confidence set (100 best models).

Variable	*Estimate*	*Unconditional SE*	*Importance*	*95%CI*
Forest birds				
(Intercept)	-3.315	5.513	1	-14.5 to 7.875
NDVI_mn_SU	41.273	7.113	1	26.84 to 55.71
RadRg	0.0365	0.015	0.985	0.007 to 0.066
NDVI_var9x9_sd_SP	-0.0089	0.015	0.336	-0.038 to 0.02
Largest Patch_OF	0.0363	0.069	0.248	-0.104 to 0.176
NDVI_var3x3_sd_OF_SP	-0.0058	0.011	0.243	-0.028 to 0.017
I(%WterAr^2)	0.0064	0.014	0.204	-0.021 to 0.034
%WterAr	0.0052	0.063	0.148	-0.123 to 0.133
NDVI_mn_SP	-1.2834	3.263	0.128	-7.908 to 5.341
%UrbnAr	0.0397	0.112	0.105	-0.188 to 0.267
NDVI_ent3x3_mn_OF_SU	-0.2198	0.648	0.092	-1.536 to 1.096
NDVI_ent9x9_sd_OF_SP	-0.2178	0.668	0.091	-1.572 to 1.137
TminA	-0.0073	0.074	0.079	-0.158 to 0.144
%OthFor	0.0012	0.009	0.074	-0.018 to 0.021
NDVI_ent3x3_sd_SP	0.134	1.090	0.067	-2.078 to 2.346
Open-land birds				
(Intercept)	39.975	23.979	1	-8.744 to 88.69
NDVI_var9x9_sd_OP_SP	-0.045	0.013	1	-0.072 to -0.018
NDVI_ent3x3_sd_SU	27.129	7.991	0.993	10.89 to 43.36
NDVI_mn_SP	-37.874	24.021	0.809	-86.66 to 10.92
NDVI_ent9x9_mn_OP_SP	-0.955	1.045	0.584	-3.078 to 1.167
NDVI_var9x9_mn_OP_SP	-0.002	0.003	0.296	-0.009 to 0.005
NDVI_var3x3_mn_OP_SU	0.002	0.004	0.293	-0.006 to 0.01
NDVI_sd_SP	12.264	22.847	0.284	-34.14 to 58.67
TmeanT	0.175	0.606	0.173	-1.057 to 1.406
%WterAr	-0.012	0.057	0.152	-0.128 to 0.104
%UrbnAr	0.019	0.126	0.151	-0.237 to 0.275
All species				
(Intercept)	65.127	49.598	1	-35.57 to 165.8
RadRg	0.050	0.046	0.693	-0.043 to 0.143
NDVI_var3x3_mn_SU	0.017	0.016	0.648	-0.016 to 0.051
I(Elev_mn^2)	0.000	0.000	0.454	-0.0001 to 0.0003
NDVI_mn_SP	-23.248	31.780	0.426	-87.69 to 41.19
TmaxJ	-1.045	1.574	0.349	-4.239 to 2.149
NDVI_sd_SU	13.955	24.125	0.259	-35.02 to 62.93
Elev_mn	-0.015	0.032	0.244	-0.079 to 0.049
NDVI_sd_SP	9.880	19.391	0.157	-29.49 to 49.25
%OpnAr	-0.006	0.014	0.106	-0.034 to 0.021
%UrbnAr	0.072	0.187	0.065	-0.306 to 0.451
AnPrecip_mn	-0.002	0.004	0.063	-0.009 to 0.007
NDVI_ent9x9_mn_SP	-0.157	0.526	0.049	-1.224 to 0.911
%OthFor	0.004	0.014	0.039	-0.025 to 0.033

Supplementary Table S3. Full list of observed species, species habitat preference, and number of cells (N cells) in which they were present. Grey shadowed lines represent the species present in at least 5 cells, used in the correspondence analysis. Habitat group: 1 – Farmland specialists, 2 – Farmland generalists, 3 – Forest specialists, 4 – Forest generalists, 5 – Edge species and 6 – Special elements. The conservation status is also shown for the IUCN Red List and for the Portuguese Red List (LVVP).

Scientific Name	*Code*	*Habitat group*	*LVVP*	*IUCN*	*Ncells*
Acrocephalus arundinaceus (Linnaeus, 1758)	acraru	6	LC	LC	7
Acrocephalus scirpaceus (Hermann, 1804)	acrsci	6	NT	LC	3
Aegithalos caudatus (Linnaeus, 1758)	aegcau	4	LC	LC	6
Alauda arvensis (Linnaeus, 1758)	alaarv	1	LC	LC	1
Alcedo atthis (Linnaeus, 1758)	alcatt	6	LC	LC	3
Alectoris rufa (Linnaeus, 1758)	aleruf	5	LC	LC	27
Anthus campestris (Linnaeus, 1758)	antcam	1	LC	LC	2
Anthus pratensis (Linnaeus, 1758)	antpra	2	LC	LC	2
Calandrella brachydactyla (Leisler, 1814)	calbra	1	LC	LC	2
Carduelis cannabina (Linnaeus, 1758)	carcan	5	LC	LC	25
Carduelis carduelis (Linnaeus, 1758)	carcar	5	LC	LC	37
Certhia brachydactyla (C. L. Brehm, 1820)	cerbra	4	LC	LC	26
Cettia cetti (Temminck, 1820)	cetcet	6	LC	LC	20
Chloris chloris (Linnaeus, 1758)	chlchl	4	LC	LC	32
Cisticola juncidis (Rafinesque, 1810)	cisjun	2	LC	LC	40
Clamator glandarius (Linnaeus, 1758)	clagla	5	VU	LC	6
Coccothraustes coccothraustes (Linnaeus, 1758)	coccoc	3	LC	LC	4
Columba livia (Gmelin, 1789)	colliv	5	DD	LC	16
Columba palumbus (Linnaeus, 1758)	colpal	4	LC	LC	15
Coracias garrulus (Linnaeus, 1758)	corgar	2	CR	LC	1
Coturnix coturnix (Linnaeus, 1758)	cotcot	2	LC	LC	26
Cuculus canorus (Linnaeus, 1758)	cuccan	4	LC	LC	30
Cyanistes caeruleus (Linnaeus, 1758)	cyacae	4	LC	LC	36
Cyanopica cooki (Bonaparte, 1850)	cyacok	5	LC	LC	23
Dendrocopos major (Linnaeus, 1758)	denmaj	4	LC	LC	19
Dendrocopos minor (Linnaeus, 1758)	denmin	3	LC	LC	9
Emberiza calandra (Linnaeus, 1758)	embcal	2	LC	LC	40
Emberiza cia (Linnaeus, 1766)	embcia	5	LC	LC	1
Emberiza cirlus (Linnaeus, 1766)	embcir	5	LC	LC	15
Erithacus rubecula (Cuvier, 1800)	erirub	3	LC	LC	3
Fringilla coelebs (Linnaeus, 1758)	fricoe	4	LC	LC	36
Galerida cristata (Linnaeus, 1758)	galcri	1	LC	LC	33
Galerida theklae (A. E. Brehm, 1857)	galthe	2	LC	LC	12
Garrulus glandarius (Linnaeus, 1758)	gargla	4	LC	LC	20
Hippolais polyglotta (Vieillot, 1817)	hippol	5	LC	LC	23
Jynx torquilla (Linnaeus, 1758)	jyntor	5	DD	LC	2
Lanius meridionalis (Temminck, 1820)	lanmer	5	LC	LC	27
Lanius senator (Linnaeus, 1758)	lansen	5	NT	LC	28
Lophophanes cristatus (Linnaeus, 1758)	lopcri	4	LC	LC	12
Lullula arborea (Linnaeus, 1758)	lularb	5	LC	LC	34

Luscinia megarhynchos (C. L. Brehm, 1831)	lusmeg	4	LC	LC	34
Melanocorypha calandra (Linnaeus, 1766)	melcal	1	NT	LC	2
Merops apiaster (Linnaeus, 1758)	merapi	2	LC	LC	38
Monticola solitarius (Linnaeus, 1758)	monsol	6	LC	LC	2
Motacilla alba (Linnaeus, 1758)	motalb	6	LC	LC	9
Motacilla cinerea (Tunstall, 1771)	motcin	6	LC	LC	3
Motacilla flava (Linnaeus, 1758)	motfla	6	LC	LC	1
Oenanthe hispanica (Linnaeus, 1758)	oenhis	2	VU	LC	4
Oriolus oriolus (Linnaeus, 1758)	oriori	4	LC	LC	20
Otis tarda (Linnaeus, 1758)	otitar	1	EN	VU	3
Parus major (Linnaeus, 1758)	parmaj	4	LC	LC	36
Passer domesticus (Linnaeus, 1758)	pasdom	2	LC	LC	38
Passer hispaniolensis (Temminck, 1820)	pashis	2	LC	LC	12
Passer montanus (Linnaeus, 1758)	pasmon	5	LC	LC	8
Petronia petronia (Linnaeus, 1766)	petpet	5	LC	LC	14
Phoenicurus ochruros (S. G. Gmelin, 1774)	phooch	6	LC	LC	3
Phoenicurus phoenicurus (Linnaeus, 1758)	phopho	3	LC	LC	7
Phylloscopus bonelli (Vieillot, 1819)	phybon	3	LC	LC	3
Phylloscopus ibericus (Ticehurst, 1937)	phyibe	3	LC	LC	11
Pica pica (Linnaeus, 1758)	picpic	5	LC	LC	12
Picus viridis (Linnaeus, 1758)	picvir	5	LC	LC	8
Pterocles orientalis (Linnaeus, 1758)	pteori	1	EN	LC	2
Serinus serinus (Linnaeus, 1766)	serser	4	LC	LC	34
Sitta europaea (Linnaeus, 1758)	siteur	3	LC	LC	26
Saxicola rubicola (Linnaeus, 1766)	srubi	2	LC	LC	37
Streptopelia decaocto (Frivaldszky, 1838)	strdec	4	LC	LC	38
Streptopelia turtur (Linnaeus, 1758)	strtur	5	LC	LC	11
Sturnus unicolor (Temminck, 1820)	stuuni	2	LC	LC	39
Sylvia atricapilla (Linnaeus, 1758)	sylatr	3	LC	LC	14
Sylvia cantillans (Pallas, 1764)	sylcan	5	LC	LC	3
Sylvia hortensis (Gmelin, 1789)	sylhor	5	NT	LC	2
Sylvia melanocephala (Gmelin, 1789)	sylmel	4	LC	LC	33
Sylvia undata (Boddaert, 1783)	sylund	5	LC	NT	3
Tetrax tetrax (Linnaeus, 1758)	tettet	1	VU	NT	8
Troglodytes troglodytes (Linnaeus, 1758)	trgtrg	3	LC	LC	21
Turdus merula (Linnaeus, 1758)	turmer	4	LC	LC	37
Turdus viscivorus (Linnaeus, 1758)	turvis	4	LC	LC	8
Upupa epops (Linnaeus, 1758)	upuepo	5	LC	LC	35

Supplementary Table S4. Total list of well-surveyed cells in the 2010-2012 time window (n= 91 cells). From this list, only 40 cells (shadowed in grey) were selected for data analysis after intersecting with the 59 cells with adequate land cover data. (N – number of records; S – observed species richness)

	Observed		***Estimated species richness***			***%Completeness***		
**Cell**	**N**	**S**	**Mao Tau 95% CI Upper Bound**	**Chao 2 Mean**	**Jack 1 Mean**	**Mao Tau**	**Chao2**	**Jackknife1**
ey9v7	273	38	41.55	40.49	43.98	91%	94%	86%
ey9ve	222	41	44.3	43.32	47.97	93%	95%	85%
ey9vk	161	35	38.69	37.49	41.96	93%	96%	86%
ey9zv	139	29	31.03	29.6	31.98	93%	98%	91%
eycb5	522	47	52.38	51.99	52.99	90%	90%	89%
eycb7	63	24	26.23	24.98	28.92	91%	96%	83%
eycbh	350	42	46.53	46.19	48.98	90%	91%	86%
eycbj	204	39	43.96	43.48	47.96	91%	92%	83%
eycbq	88	21	25.9	25.94	26.93	81%	81%	78%
eycbw	217	33	35.21	33.85	36.98	94%	97%	89%
eycbx	142	27	31.33	30.72	32.96	86%	88%	82%
eycbz	55	21	23.02	21.74	24.93	91%	97%	84%
eycc9	147	33	34.96	33.76	37.97	94%	98%	87%
eyccd	100	21	24.22	22.98	25.95	87%	91%	81%
eycfs	955	50	57.16	57.59	59.99	91%	90%	87%
eycsv	204	48	57.97	63.94	63.94	88%	80%	80%
eyctz	884	41	45.48	44.75	46.99	90%	92%	87%
eycvc	1022	46	51.16	51.24	52.99	90%	90%	87%
eycvn	173	35	38.74	37.98	41.96	90%	92%	83%
eycvp	346	42	45.95	44.99	47.98	91%	93%	88%
eycvr	139	29	32.5	31.48	34.96	89%	92%	83%
eycwr	753	48	51.36	50	52.99	93%	96%	91%
eycww	190	34	38.2	37.98	41.96	89%	90%	81%
eycwz	409	37	43.65	48.64	45.98	87%	78%	83%
eycxj	70	24	28.78	29.52	31.89	83%	81%	75%
eycxq	153	29	32.22	31.13	34.96	90%	93%	83%
eycxr	69	24	28.38	28.6	31.88	85%	84%	75%
eycy2	56	22	26.26	26.13	28.88	84%	84%	76%
eycy3	185	39	44.52	46.46	48.95	88%	84%	80%
eycy6	133	37	43.52	45.8	48.92	87%	83%	78%

eycy8	221	40	45.96	49.13	50.95	87%	81%	79%
eycz1	122	30	33.89	33.47	37.93	89%	90%	79%
eycz3	287	39	44.66	45.98	46.97	87%	85%	83%
eycz4	121	26	29.83	28.98	31.95	87%	90%	81%
eycz6	478	38	42.46	41.74	43.99	89%	91%	86%
eyczc	1168	40	43.1	41.5	44	93%	96%	91%
eyczg	146	31	37.4	41.93	41.93	86%	76%	76%
eyczn	104	22	25.59	23.98	25.96	86%	92%	85%
eyczp	194	30	34.41	34.18	36.96	87%	88%	81%
eyczq	158	31	35.93	36.56	38.95	86%	85%	80%
eyczu	349	36	38.83	37.5	39.99	95%	99%	93%
eyczw	174	32	36.95	37.57	39.95	87%	85%	80%
eydjc	126	37	42.9	46.09	47.91	86%	80%	77%
eydkg	294	41	47.74	52.21	50.97	86%	79%	80%
eydn0	155	35	39.11	38.97	43.94	89%	90%	80%
eydn1	141	36	40.66	41.11	44.94	89%	88%	80%
eydn4	70	25	28.19	27.11	30.91	89%	92%	81%
eydp5	73	22	26.76	27.18	28.9	82%	81%	76%
eydp7	199	38	42.8	43.6	47.95	89%	87%	79%
eydph	236	32	38.39	41.29	39.97	83%	78%	80%
eydps	129	31	35.42	35.17	37.95	88%	88%	82%
eydpt	106	31	34.46	33.6	37.93	90%	92%	82%
eydq2	160	47	55.18	62.91	62.91	87%	76%	76%
eydq3	41	22	22.94	22.15	24.93	96%	99%	88%
eydq8	115	36	40.21	39.97	43.93	90%	90%	82%
eyf30	198	38	40.97	39.9	44.96	93%	95%	85%
eyf31	283	35	39.43	38.74	40.98	89%	90%	85%
eyf33	85	24	26.36	25.1	28.94	91%	96%	83%
eyf4c	124	30	31.92	30.66	33.97	94%	98%	88%
eyf4g	54	27	27	27	27	100%	100%	100%
eyf51	191	34	39.02	39.22	40.96	87%	87%	83%
eyf53	205	34	37.53	36.49	39.97	91%	93%	85%
eyf56	257	34	38.42	37.74	39.98	88%	90%	85%
eyf5m	97	31	36.08	35.96	40.91	89%	89%	78%

eyf5n	166	38	43.05	43.96	46.95	88%	86%	81%
eyf5q	228	50	55.65	59.96	54.98	90%	83%	91%
eyf5r	85	32	37.39	39.41	41.88	86%	81%	76%
eyf6y	105	30	34.01	33.47	36.93	88%	90%	81%
eyf7n	375	38	45.12	49.97	46.98	84%	76%	81%
eyf7p	342	39	43.08	42.49	45.98	91%	92%	85%
eyf7r	231	33	37.45	37.18	39.97	88%	89%	83%
eyf9b	75	22	23.03	22.12	23.97	96%	99%	92%
eyfd1	80	33	35.95	34.98	40.9	92%	94%	81%
eyfd2	99	30	32.82	31.65	35.94	91%	95%	83%
eyfd3	84	28	32.13	31.95	35.9	87%	88%	78%
eyfdb	285	37	43.93	50.06	48.96	87%	76%	78%
eyfe3	171	32	36.18	35.98	39.95	88%	89%	80%
eyfh8	268	38	40.7	39.2	41.99	93%	97%	90%
eyfh9	132	33	38.55	39.95	40.94	86%	83%	81%
eyfhb	182	32	36.43	36.18	38.96	88%	88%	82%
eyfhc	389	41	44.74	42.99	44.99	92%	95%	91%
eyfhf	165	27	29.67	28.19	30.98	91%	96%	87%
eyfhs	231	34	38.08	36.49	38.98	92%	96%	90%
eyfj2	64	22	25.76	24.95	27.91	85%	88%	79%
eyfp0	82	24	27.45	26.47	29.93	87%	91%	80%
eyfq7	62	24	28.07	27.94	31.87	86%	86%	75%
eyfu1	65	24	25.36	24.3	26.95	95%	99%	89%
eyfxc	376	44	46.07	44.75	47.99	96%	98%	92%
eyfy4	97	27	30.21	29.12	32.94	89%	93%	82%
eyfy7	98	27	30.67	29.97	33.93	88%	90%	80%
eyfyk	114	25	29.81	30.55	32.93	84%	82%	76%

Supplementary Table S5. Observed richness of species groups in the 40 cells selected for data analyses.

Cell	*Open-land birds*	*Forest birds*	*All species*
**ey9vk**	17	18	35
**ey9zv**	15	14	29
**eycb5**	23	24	47
**eycb7**	8	16	24
**eycbh**	20	22	42
**eycbj**	21	18	39
**eycbq**	12	9	21
**eycbw**	17	16	33
**eycbx**	16	11	27
**eycfs**	26	24	50
**eycsv**	28	20	48
**eycy6**	18	19	37
**eycy8**	18	22	40
**eyczu**	22	14	36
**eydkg**	24	17	41
**eydp5**	16	6	22
**eydp7**	25	13	38
**eydq3**	18	4	22
**eyf31**	21	14	35
**eyf33**	18	6	24
**eyf4g**	19	8	27
**eyf51**	18	16	34
**eyf53**	20	14	34
**eyf56**	20	14	34
**eyf5m**	17	14	31
**eyf5n**	22	16	38
**eyf5q**	26	24	50
**eyf5r**	13	19	32
**eyf6y**	19	11	30
**eyf7n**	27	11	38
**eyfd3**	18	10	28
**eyfdb**	22	15	37
**eyfe3**	19	13	32
**eyfhf**	13	14	27
**eyfhs**	22	12	34
**eyfq7**	10	14	24
**eyfu1**	13	11	24
**eyfy4**	17	10	27
**eyfy7**	17	10	27
**eyfyk**	18	7	25
